# SARS-CoV-2-triggered mast cell rapid degranulation induces alveolar epithelial inflammation and lung injury

**DOI:** 10.1038/s41392-021-00849-0

**Published:** 2021-12-17

**Authors:** Meng-Li Wu, Feng-Liang Liu, Jing Sun, Xin Li, Xiao-Yan He, Hong-Yi Zheng, Yan-Heng Zhou, Qihong Yan, Ling Chen, Guo-Ying Yu, Junbiao Chang, Xia Jin, Jincun Zhao, Xin-Wen Chen, Yong-Tang Zheng, Jian-Hua Wang

**Affiliations:** 1 College of Life Science, Henan Normal University, Xinxiang, 453007 China; 2 Key Laboratory of Animal Models and Human Disease Mechanisms of the Chinese Academy of Sciences, Kunming Institute of Zoology, Chinese Academy of Sciences, Kunming, 650223 China; 3 State Key Laboratory of Respiratory Disease, National Clinical Research Center for Respiratory Disease, Guangzhou Institute of Respiratory Health, The First Affiliated Hospital of Guangzhou Medical University, Guangzhou, Guangdong, 510182 China; 4 Guangzhou Institutes of Biomedicine and Health, Chinese Academy of Sciences, Guangzhou, 510530 China; 5 Bioland Laboratory, Guangzhou Regenerative Medicine and Health Guangdong Laboratory, Guangzhou, 510005 China; 6 Shanghai Public Health Clinical Center Affiliated to Fudan University, Shanghai, 201508 China; 7 University of Chinese Academy of Sciences, Beijing, 100039 China

**Keywords:** Microbiology, Infectious diseases

## Abstract

SARS-CoV-2 infection-induced hyper-inflammation links to the acute lung injury and COVID-19 severity. Identifying the primary mediators that initiate the uncontrolled hypercytokinemia is essential for treatments. Mast cells (MCs) are strategically located at the mucosa and beneficially or detrimentally regulate immune inflammations. In this study, we showed that SARS-CoV-2-triggered MC degranulation initiated alveolar epithelial inflammation and lung injury. SARS-CoV-2 challenge induced MC degranulation in ACE-2 humanized mice and rhesus macaques, and a rapid MC degranulation could be recapitulated with Spike-RBD binding to ACE2 in cells; MC degranulation altered various signaling pathways in alveolar epithelial cells, particularly, the induction of pro-inflammatory factors and consequential disruption of tight junctions. Importantly, the administration of clinical MC stabilizers for blocking degranulation dampened SARS-CoV-2-induced production of pro-inflammatory factors and prevented lung injury. These findings uncover a novel mechanism for SARS-CoV-2 initiating lung inflammation, and suggest an off-label use of MC stabilizer as immunomodulators for COVID-19 treatments.

## Abstract (web summary)

A schematic illustration of SARS-CoV-2 triggers MC rapid degranulation to induce alveolar epithelial inflammation and lung injury. SARS-CoV-2 triggers an immediate mast cell (MC) degranulation, which initiates the alveolar epithelial inflammation and disrupts the tight junction. MC stabilizers that block degranulation reduce virus-induced lung inflammation and injury.

## Graphical abstract

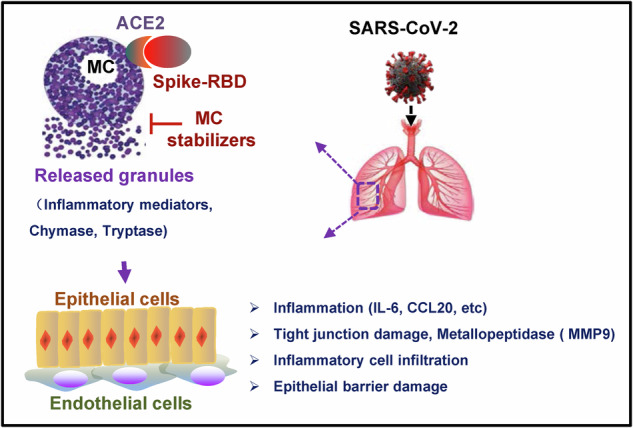

## Introduction

The Coronavirus disease 2019 (COVID-19) caused by severe acute respiratory syndrome coronavirus 2 (SARS-CoV-2) has become a global pandemic. A higher incidence of severity and deaths in older individuals has been observed.^1^ To curtail this disease, the nucleotide analogue, Remdesivir (Veklury), has been approved by U.S Food and Drug Administration (FDA) for treating COVID-19 in adults and older chilren (more than 12 years of age);^2^ another ribonucleoside analogue, molnupiravir (EIDD-2801, MK-4482), developed by Merck and Ridgeback, was recently approved by United Kingdom for oral treatment of COVID-19.^3,4^ Besides, US FDA has approved and authorized two mRNA vaccines (Pfizer-BioNTech, Moderna) and one recombinant adenovirus-26 vaccine (Janssen, Ad26.COV2.S) for population use, and globally more than 100 candidate SARS-CoV-2 vaccines are under development.^5^

A key pathologic feature of COVID-19 is the hyper-inflammatory response (also termed hypercytokinemia or “cytokine storm”) in association with severe COVID-19 disease, and those inflammatory cytokines and chemokines produced in vivo lead to damage to the alveolar epithelial cells and capillary endothelial cells.^6–14^ Known inflammatory cytokines and chemokines, which are excessively produced in patients with severe COVID-19, include IL-6, IL-8, IL-1β, TNF-α, IFN-γ, MIP1α and 1β, CCL2, CCL5, CCL20, CXCL1, CXCL2, CXCL8, CXCL10 and CXCL17.^8,12–19^ Among them, elevated IL-6, TNF-α and C-reaction protein (CRP) levels have been shown to be independent risk factors for the severity of COVID-19 disease.^12,16,19–21^

Therefore, targeting the hyper-inflammation loop to develop the immunomodulatory agents is an attractive strategy for developing treatment for severe COVID-19 cases.^6,22,23^ Some drug candidates have been tried in order to mitigate hyper-inflammation and improve clinical outcomes. These include humanized anti-IL-6 receptor antibody Tocilizumab,^24^ anti-IFN-γ monoclonal antibody Emapalumab, IL-1 receptor antagonist Anakinra (NCT04324021), JAK1 and JAK2 inhibitors Baricite and Rituxolitinib^25^ (NCT04338958), and the Corticosteroids such as Dexamethasone.^26–28^ However, Tocilizumab was not effective for preventing intubation or death in moderately ill hospitalized patients;^29,30^ another IL-6 receptor monoclonal antibodies Sarilumab not only failed to improve clinic outcomes and reduce mortality, but led to serious complications.^31^ Of note, despite the probable benefit of calming the cytokine storm, suppressing inflammation and immune response may impede on viral clearance.^32^ Indeed, corticosteroid treatment suppresses the overall immune responses, but also impairs the induction of anti-viral type-I interferon responses.^33,34^ Therefore, the development of effective immunomodulatory agents that suppress inflammation, without compromising host immune protection, is required.

Lung is the primary target for the corona viruses. The alveoli of the lung are covered with angiotensin-converting enzyme 2 (ACE2)‐expressing epithelial cells. It is reasonable to hypothesis that the alveoli epithelial inflammation is a prerequisite for damage of both alveoli epithelial cells and capillary endothelial cells during SARS-CoV-2 infection,^35–37^ during which infected epithelial cells recruit and activate monocytes and macrophages to secrete pro-inflammatory cytokines, which further recruits neutrophils and activates T-cells to exacerbate inflammation.^11^ As shown in the postmortem lung biopsies, there were diffuses alveolar damage with necrosis of alveolar lining cells, pneumocyte type 2 hyperplasia, and linear intra-alveolar fibrin deposition, widespread vascular thrombosis with microangiopathy and occlusion of alveolar capillaries.^38,39^ Therefore, uncovering the mechanisms of epithelial inflammation caused SARS-CoV-2 is of uppermost priority.

MCs are tissue resident cells that strategically placed throughout the host-environment interface including the whole respiratory tract and the nasal cavity. Besides being as the main effector cells in allergy, MCs can interact with various immune cells through release of soluble factors or direct contact to beneficially or detrimentally regulate immune inflammations.^40–42^ A variety of pathogens including DNA/RNA virus, fungi, bacteria or their products can activate MCs, and induce secretion of cytokines and chemokines through degranulation-dependent or degranulation-independent pathways.^42^ The released mediators can drive the recruitments of imune effector cells for pathogens clearance; alternatively, these inflammatory mediators may induce inappropriate inflammatory responses to disrupt the epithelial-endothelia barriers for promoting pathogens invasion.^42^ In SARS-CoV-2 infection, the postmortem lung biopsies of COVID-19 patients show a massively increased density of perivascular and septal MCs, suggesting MCs were recruited to the alveolar septa to play certain unknown functions.^43,44^ SARS-CoV-2 infection triggered MC infiltration into the pulmonary parenchyma of african green monkeys.^45^ These evidences indicate a potential role of MCs in SARS-CoV-2 pathogenesis.

Therefore, in this study, we investigated the role of MCs in SARS-CoV-2 infection, and examined whether SARS-CoV-2 could induce MC degranulation and its sequential role in virus-induced alveolar epithelial inflammation and lung injury, and we hope to find the potent immunomodulators to treat COVID-19.

## Results

### SARS-CoV-2 induces MC degranulation in lung of ACE2-humanized mice

**Fig. 1:**
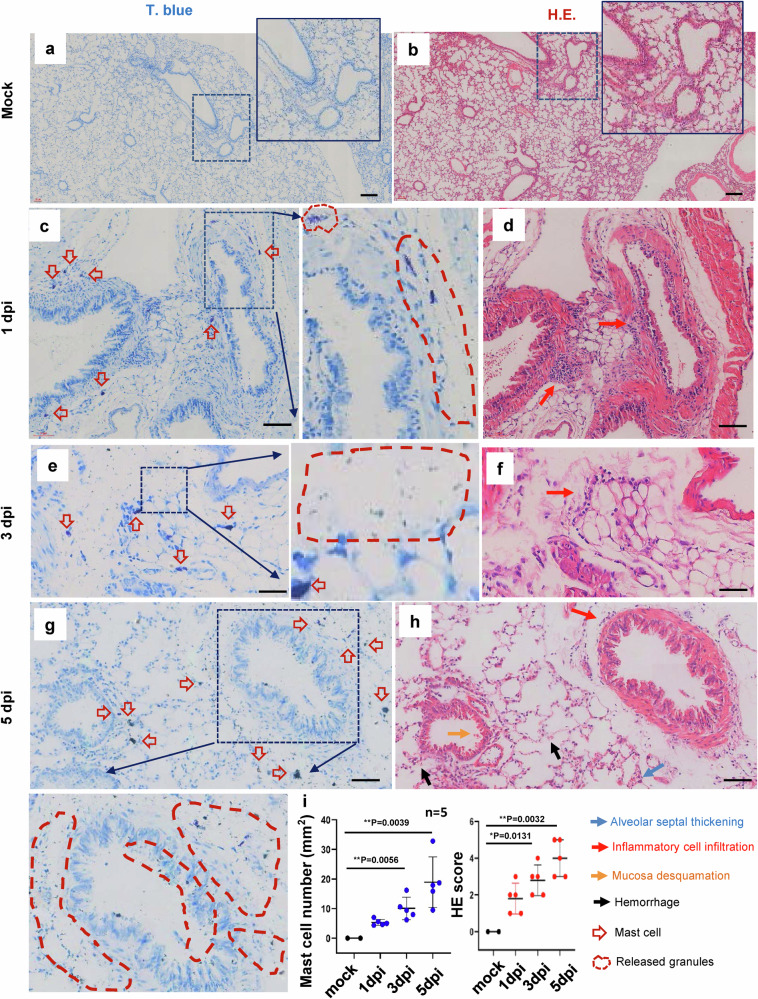
SARS-CoV-2 induces mast cell degranulation and lung injury in hACE2-humanized mice. Five C57BL/6N-Ace2^em2(hACE2-WPRE,pgk-puro)/CCLA^ mice were intranasally infected with SARS-CoV-2 (strain 107) at a dose of 2 × 10^6^ TCID_50_, two mice were used as the mock-infections. The mice were euthanized at the 1 dpi, 3 dpi and 5 dpi, and the lung tissues were harvested for histological analysis. Toluidine blue staining was used to observe MCs and their degranulation (**a**, **c**, **e** and **g**), and the lung injury was observed by H.E. staining (**b**, **d**, **f** and **h**), scale bar: 100 μm. (i) The pathological score was assessed according to the degree of lung tissue lesions and MC count in lung sections was calculated. *p < 0.05 and **p < 0.01 are considered significant differences

To investigate whether SARS-CoV-2 could induce MC degranulation in vivo, the ACE2-humanized inbred mice,^46^ termed C57BL/6N-Ace2^em2(hACE2-WPRE,pgk-puro)/CCLA^, were intranasally infected with SARS-CoV-2 (strain 107) at a dose of 2 × 10^6^ TCID_50_ (50% tissue culture infective dose), then euthanized at the different days post-infection (dpi) to harvest lung tissues for histological analysis. The same amount of PBS was inoculated as the mock infection. Our results confirmed previous observation that SARS-CoV-2 virions were mainly distributed in the peri-bronchus and bronchioalveolar-duct junction,^46–48^ as shown by immunostaining of SARS-CoV-2 nucleocapsid protein at 1- and 3-dpi (Supplementary Fig. 1). MCs and their degranulation are indicated by the metachromatic labeling with Toluidine blue (T. blue).^49^ Compared with mock-infection (Fig.1a), at the 1 dpi, the accumulation of MCs in peri-bronchus was observed and the release of granules was found in the alveolar space (Fig. 1c). The MC degranulation over the course of SARS-CoV-2 infection could be seen with abundance of released granules distributed widely in alveolar spaces (Fig. 1c, e, g). The lung lesions around the area of MC accumulation and degranulation were examined by staining with Hematoxylin and eosin (H.E.). Compared with mock-infection control (Fig. 1b), lung lesions, including inflammatory cells (lymphocytes and monocytes) infiltration, hemorrhage, alveolar septal thickening, and mucosa desquamation, were observed around the areas of MC accumulation and degranulation (Fig. 1d, f, h). The pathological score was assessed according to the degree of lung tissue lesions and MC count in lung sections was calculated (Fig. 1i).

To ensure the above observations are not limited to one experimental model of SARS-CoV-2 infection, another mouse model, namely Ad5-hACE2-transduced BALB/c mice,^50^ were used. The mice were intranasal challenge with 7 × 10^4^ TCID_50_ SARS-CoV-2, then sacrificed at different times to harvest the lungs for histological staining. Similar results were obtained. Compared with the mock-infection (Supplementary Fig. 2a), SARS-CoV-2 challenge induced a MC degranulation in the peri-bronchus and bronchioalveolar-duct junction (Supplementary Fig. 2c, e, g). Simultaneous H.E. staining of the adjacent lung section showed lung lesions around these areas (Supplementary Fig. 2d, f, h), but not in mock-infected mice (Supplementary Fig. 2b). The pathological score was assessed according to the degree of lung tissue lesions and MC count in lung sections was calculated (Supplementary Fig. 2i). Taken together, these data demonstrate convincingly that SARS-CoV-2 infection induces MC accumulation and degranulation around the area of lung lesion in ACE2-humanized mice.

### SARS-CoV-2 induces MC degranulation in lung of rhesus macaque

Having firmly established a pathogenesis model in mice, we went on the investigation whether it was producible in nonhuman primate, which has often been considered as the translational gate-keeper before any scientific discovery is progressed to human testing. The SARS-CoV-2 can infect Chinese rhesus macaques (chRMs) (*Macaca mulatta*), in which much of the characteristics of immunological pathogenesis of human COVID-19 can be recapitulated; notably, aged chRMs showed the delayed but more severe cytokine storm and higher immune cell infiltration, compared with young chRMs.^51^ This model is therefore most suited for in-depth dissection of inflammatory responses in relation to MC activation.

**Fig. 2:**
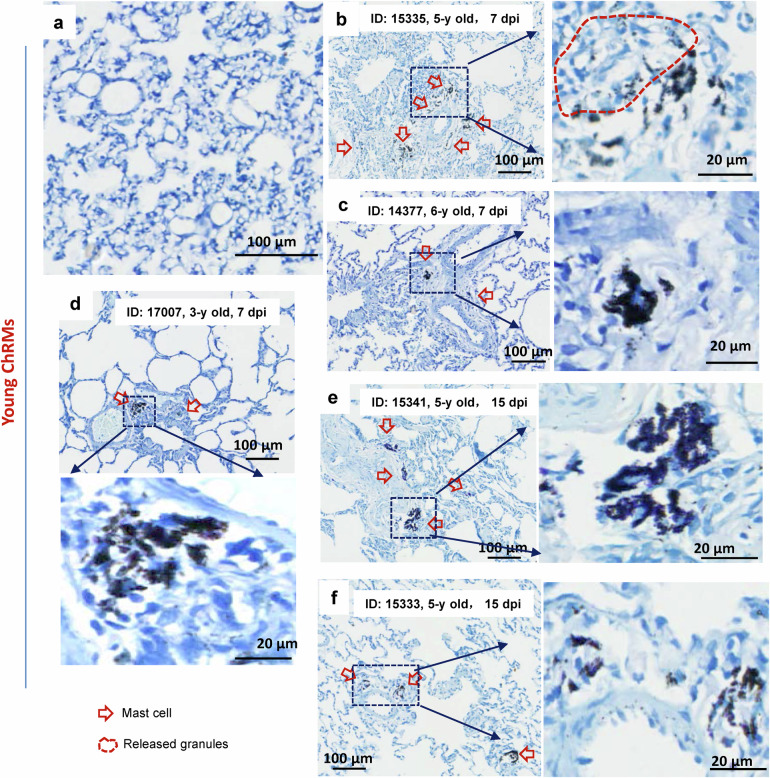
SARS-CoV-2 induces mast cell degranulation in young chRMs. Young chRMs (3- to 6-year old) were anaesthetized by Zoletil 50 and intratracheally inoculated with SARS-CoV-2 (1 × 10^7^ TCID_50_) in a 2 mL volume by bronchoscope. **a** Mock infection. The animals were euthanized at 7 dpi (**b**, **c**, **d**) or 15 dpi (**e**, **f**) and the lung lobes were collected for histology analysis. Toluidine blue staining was used to observe MCs and their degranulation. Red arrow indicates the MCs

**Fig. 3:**
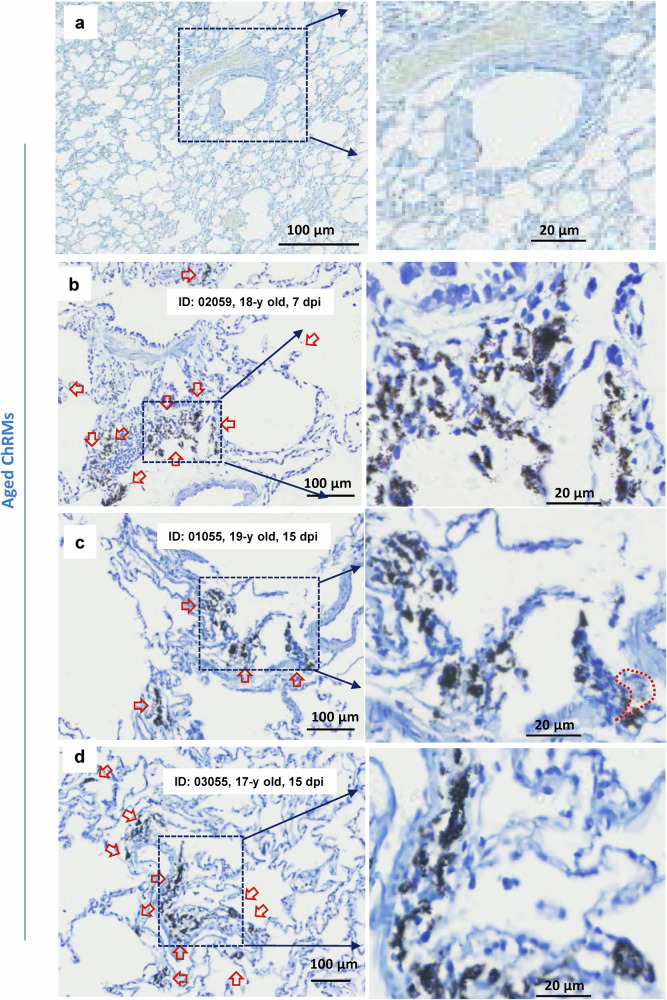
SARS-CoV-2 induces mast cell degranulation in aged chRMs. Aged chRMs (17- to 19-year old) were anaesthetized by Zoletil 50 and intratracheally inoculated with SARS-CoV-2 (1 × 10^7^ TCID_50_) in a 2 mL volume by bronchoscope. **a** Mock infection. The animals were euthanized at 7 dpi (**b**) or 15 dpi (**c**, **d**) and the lung lobes were collected for histology analysis. Toluidine blue staining was used to observe MCs and their degranulation. Red arrow indicates the MCs

We confirmed that SARS-CoV-2 infection led to MC degranulation in lungs of chRMs. The young chRMs (3- to 6-year old) (Fig. 2) or aged chRMs (17- to 19-year old) (Fig. 3) were anesthetized, then intratracheally infected with SARS-CoV-2 (1 × 10^7^ TCID_50_) (strain 107). The animals were sacrificed at 7 or 15 dpi, and lungs were harvested and sectioned for T. blue staining to examine changes in MCs. Compared with the mock-infections (Fig. 2a, 3a), SARS-CoV-2 challenge recruited MCs accumulation in the peri-bronchus and bronchioalveolar-duct junction at both 7- and 15- dpi, with noted MC degranulation (Figs. 2b–f, 3b–d). As expected, compared with young chRMs (Fig. 2b–f), aged chRMs have showed more extensive SARS-CoV-2-induced MCs accumulation and degranulation (Fig. 3b–d). Taken together, we demonstrate that SARS-CoV-2 induces MC degranulation in lung of rhesus macaque.

### The binding of Spike-RBD to ACE2 triggers a rapid MC degranulation

**Fig. 4:**
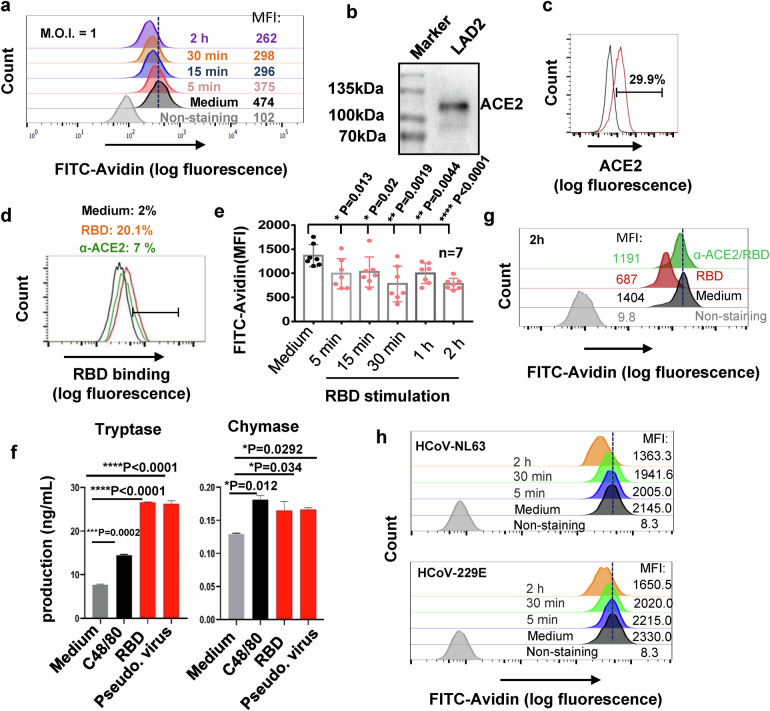
The binding of Spike-RBD to ACE2 triggers a rapid MC degranulation. **a** SARS-CoV-2 (M.O.I. = 1)-induced LAD2 degranulation, detected by flow cytometry with immunostaining the intracellular avidin. **b**, **c** ACE2 expression in LAD2 cells, detected with Western blotting and flow cytometry with immunostaining with specific antibodies. **d** Prior-blocking with anti-ACE2 antibody reduces RBD binding. LAD2 cells were incubated with anti-ACE2 antibody (5 μg/ml) at 37 °C for 1 h, then Spike-RBD (5 μg/ml) were added for binding at 4 °C for 1 h, and the binding of Spike-RBD to LAD2 cells was detected with flow cytometry. **e**, **f**, **g** Spike-RBD induces LAD2 degranulation. LAD2 cells were incubated with Spike-RBD (5 μg/ml) at 37 °C for the indicated time, then cells were fixed with 4% paraformaldehyde and permeabilized and immunostained with anti-avidin-FITC at 4 °C for 1 h, and analyzed with flow cytometry (**e**, **g**), results from 7 independent repeats were summarized and presented (**e**). The degranulated components Tryptase and Chymase were detected by ELISA (**f**). LAD2 cells were prior-treated with anti-ACE2 antibody (5 μg/ml) at 37 °C for 1 h before the Spike-RBD stimulation (**g**). **h** HCoV-NL63 and HCoV-229E induced LAD2 degranulation. LAD2 cells were incubated with HCoV-NL63 or HCoV-229E (M.O.I = 1) for the indicated time, and cell degranulation was detected as above. One representative data from 3 (**a**, **b**, **f**, **h**) or 4 (**c**, **d**, **g**) independent repeats are shown. Data are presented as mean ± SD. *p < 0.05, **p < 0.01, ***p < 0.001 and *p < 0.0001 are considered significant differences. MFI mean fluorescence intensity

To further investigate the molecular mechanism responsible for SARS-CoV-2-caused MC degranulation, we used human MC cell line LAD2 cells and first examined whether SARS-CoV-2 could induce LAD2 cell degranulation. MC degranulation can be assessed by the intensity reduction of immunostaining of cytoplasmic avidin granules as previously described.^49,52^ The cells were treated with SARS-CoV-2 (M.O.I. = 1) (strain 2019-nCoV WIV04),^53^ and results displayed that SARS-CoV-2 induced an immediate LAD2 cell degranulation, as shown by the rapid decrease of immunostaining of cytoplasmic avidin granules within 5 min of viral infection, and the induction of cellular degranulation progressed over the course of 2 h viral infection (Fig. 4a).

The observation of SARS-CoV-2-triggering a rapid MC degranulation (as short as 5 min) indicates that viral binding might be responsible for this event. LAD2 cells expressed ACE2 receptor as demonstrated by Western blotting (Fig. 4b) and flow cytometry (Fig. 4c), and were susceptible to SARS-CoV-2 and Spike-pseudotyped lentivirus (NL4-3/S) (Supplementary Fig. 3). The recombinant receptor-binding domain (RBD) of SARS-CoV-2 Spike glycoprotein could bind to LAD2 cells at 4 °C, and the prior-blocking with anti-ACE2 specific antibody prevented Spike-RBD binding (Fig. 4d). Notably, Spike-RBD binding to ACE2 was accompanied by an immediate degranulation, as shown by the significant reduction of intracellular avidin intensity within 5 min of Spike-RBD treatment (Fig. 4e, Supplementary Fig. 4a), and the degranulation was progressively increased over the course of 2 h period of incubation (Fig. 4e). Spike-RBD-induced LAD2 degranulation was also quantified by the release of intracellular Tryptase and Chymase. The treatments of LAD2 cells with either Spike-RBD or Spike-pseudotyped lentivirus (Pseudo. Virus) for 2 h significantly increased the release of Tryptase and Chymase, and the compound 48/80 (C48/80) was used as a positive experimental control for stimulation of MC degranulation (Fig. 4f).

To confirm that the specific interaction between Spike-RBD and ACE2 is key to trigger MC degranulation, we blocked the interaction by prior-treatment with anti-ACE2 antibody (Fig. 4d), and found that Spike-RBD treatment of LAD2 cells was no longer able to induce cell degranulation (Fig. 4g). The nucleocapsid protein treatment did not induce LAD2 degranulation (Supplementary Fig. 4b). A short 2 h stimulation with Spike-RBD stimulation did not show the obvious elevation of the de novo synthesis of cytokines in LAD2 cells, whereas the stimulation longer than 8 h induced productions of pro-inflammatory cytokines (Supplementary Fig. 5).

We also investigated whether other type of coronavirus could also induce MC degranulation. The coronaviruses of HCoV-NL63 and HCoV-229E were used to treat LAD2 cells (M.O.I = 1), the rapid MC degranluation was observed, as shown by the rapid decrease of immunostaining of cytoplasmic avidin granules within 5 min of viral stimulation, and the induction of cellular degranulation progressed over the course of 2 h viral infection (Fig. 4h).

Taken together, these data demonstrate that the binding of Spike-RBD of SARS-CoV-2 to ACE2 triggers an immediate MC degranulation, which is rapid and specific.

### Transcriptome analysis reveals MC degranulation-induced remodeling of cellular signalings in human alveolar epithelial cells

**Fig. 5:**
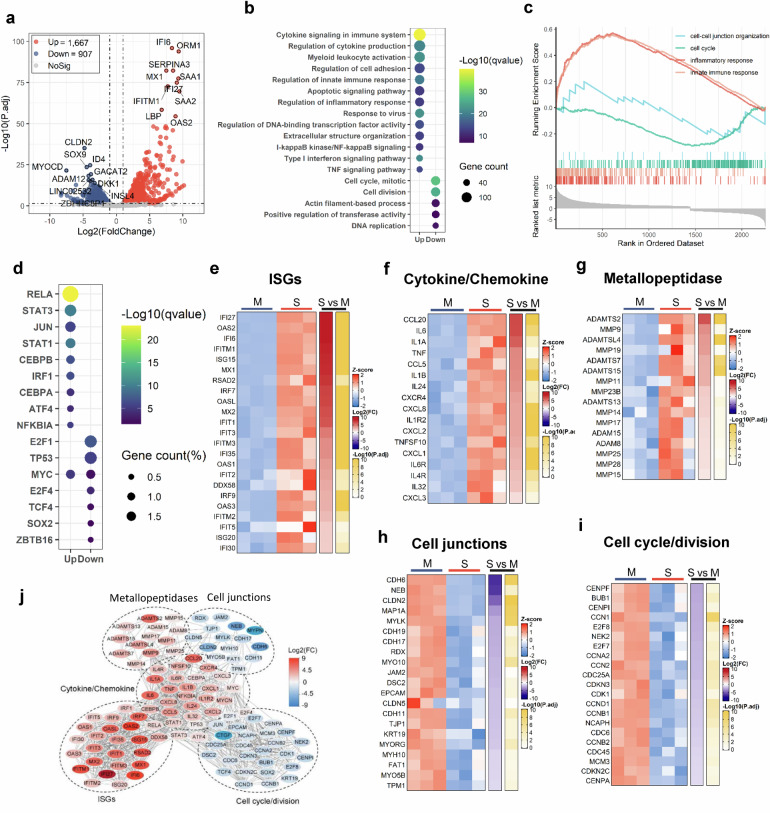
Transcriptome analysis of A549 cells treated with LAD2/RBD supernatant. **a** Volcano plot of DEGs comparing LAD2/RBD supernatant versus medium. The symbols of top 10 up-regulated or down-regulated genes are shown. **b** GO functional enrichment analysis of DEGs. The color bar indicates the minus logarithm of *q* values, and bubble size indicates the absolute gene counts enriched in a GO term. **c** GSEA showing the distribution of gene sets that related to inflammatory response, innate immune response, cell–cell junction organization or cell cycle and the enrichment scores based on DEGs. **d** Transcription-factor enrichment analysis of DEGs. The color bar indicates the minus logarithm of *q* values, and bubble size indicates the gene enrichment ratio regulated by a transcription factor. **e**–**i** Heatmaps showing relative expression level (left panel), fold change (middle panel), and adjusted *p* values (right panel) for sets of ISGs (**e**), cytokine- and chemokine-related genes (**f**), Metallopeptidase (**g**), cell junction-related genes (**h**), cell cycle- and division-related genes (**i**). M medium; S, LAD2/RBD supernatant. **j** A Protein–Protein interaction network analysis of the core DEGs. The color bar represents the fold change of protein-coded genes at transcriptome level

SARS-CoV-2 infection causes severe alveolar epithelial inflammation and barrier dysfunction.^54,55^ To test whether SARS-CoV-2-triggered MC degranulation play a role, we performed transcriptome analysis in the Spike-RBD-induced MC degranulation model. The culture supernatant of Spike-RBD-treated LAD2 cells were harvested and used to treat A549 cells (an adenocarcinomic human alveolar basal epithelial cell line) for 24 h, then the transcriptome in A549 cells were analyzed using standard protocols. Data from 3 independent repeats were summarized. The volcano plot displayed a total of 1667 up-regulated genes and 907 down-regulated genes after A549 cells were treated with supernatants from Spike-RBD-treated LAD2 cells (Fig. 5a). Gene ontology (GO) functional enrichment analysis of differently expressed genes (DEGs) showed obvious upregulation of gene sets that regulate cytokines signaling and production, the activation of myeloid and leukocyte, cell adhesion, innate immune and inflammatory response, and cell apoptotic signaling; in contrast, the gene sets that regulate cell cycle, cell division, and the actin filament-based cell movement processes were downregulated (Fig. 5b). The gene set enrichment analysis (GSEA) linked the up-regulated genes to the regulation of inflammatory and innate immune responses, and the down-regulated genes to the regulation of cell cycles and cell–cell junctions (Fig. 5c).

Transcription-factor enrichment analysis of DEGs showed that the up-regulated transcription factors were mainly those governing the immune and inflammatory response, e.g., *RELA*, *STAT3*, *STAT1*, *JUN*, *CEBP-α/β*, *IRF1* and *ATF4;* and the down-regulated transcription factors were mainly linked to the regulations of cell proliferation and metabolisms, such as *E2F1*, *E2F4*, *TP53* and *ZBTB16* (Fig. 5d).

The core DEGs with highly differential expression were catalogized. The stimulation with LAD2/RBD supernatants induced elevated expression of antiviral immune genes in A549 cells, and a significant upregulation of genes for ISGs and IFN-I responses (e.g., *IFI27*, *OAS2*, *IFI6*, *IFITM1*, *ISG15*, *MX1*, *RSAD2*, *IRF7*, *OASL*, *MX2*, *IFIT1* and *IFIT3*) (Fig. 5e) has been observed. Other prominent features were the upregulation of pro-inflammatory cytokines/chemokines, particularly *CCL20*, *IL-6*, *IL1α*, *TNF*, *CCL5*, *IL1β* and *CXCL8* (*IL-8*) genes (Fig. 5f), and an upregulation of metallopeptidase-encoding genes (e.g., *ADAMTS2* and *MMPs*) (Fig. 5g). LAD2/RBD supernatants induced a downregulation of a large number of genes encoding regulators of cell junctions and cell cycles/division. The down-regulated core DEGs linked to cell junctions included those encoding for the tight junction proteins (e.g., *CLDN2*, *CLDN5*, *TJP1* and *JAM2*), the cadherins (e.g., *CDH6*, *CDH19*, *CDH17*, *CDH11*, *FAT1*, *DSC2* and *EPCAM*), the cytoskeleton/microtubule-associated proteins (e.g., *NEB*, *KRT19*, *RDX*, *TPM1* and *MAP1A*), and myosins (e.g., *MYH10*, *MYO10*, *MYO5B*, *MYLK* and *MYORG*) (Fig. 5h). The down-regulated core DEGs linked to cell cycles/division were mainly those encoding for the mitotic Serine/Threonine kinases and their regulators (e.g., *BUB1*, *NEK2*, *CDK1; CDKN3*, *CDKN2C*), centromere proteins (e.g., *CENPF*, *CENPI*, *CENPA*), regulation of transition phase of the cell cycle related transcription factors and cyclin family members (e.g., *E2F7*, *E2F8; CCNA2*, *CCND1*, *CCNB1*, *CCNB2*), genome replication regulators (e.g., *CDC45*, *CDC6*, *MCM3*), and cell division regulators (e.g., *CCN2*, *CDC25A*), etc (Fig. 5i). A Protein–Protein interaction network (PPI) analysis of the core DEGs was constructed to visualize the relationship between DEGs and between signaling pathways (Fig. 5j).

Taken together, the transcriptome data reveal that SARS-CoV-2-triggered MC degranulation significantly alters multiple cellular signalings in human alveolar epithelial cells, particularly, MC degranulation upregulates immune responses and inflammation, and downregulates cellular signalings related to cell junction and cell division.

### Inhibition of SARS-CoV-2-triggered MC degranulation abolishes the subsequent alveolar epithelial inflammation

To seek drug candidates that may modulate MC degranulation and consequential cytokine release, we examined the known MC stabilizer Sodium cromoglicate (Sod. crom.), and the histamine receptor 1 (HR1) antagonists Ketotifen Fumarate (Ket.), Ebastine (Eba.) and Loratadine (Lor.), which are routinely used for treating human allergy. Besides of being antihistamines, Ket., Eba. (and its main metabolite carebastine) and Lor. (and its main metabolite desloratadine) can also be used as MC stabilizers to prevent degranulation and release of inflammatory mediators.^56–62^

**Fig. 6:**
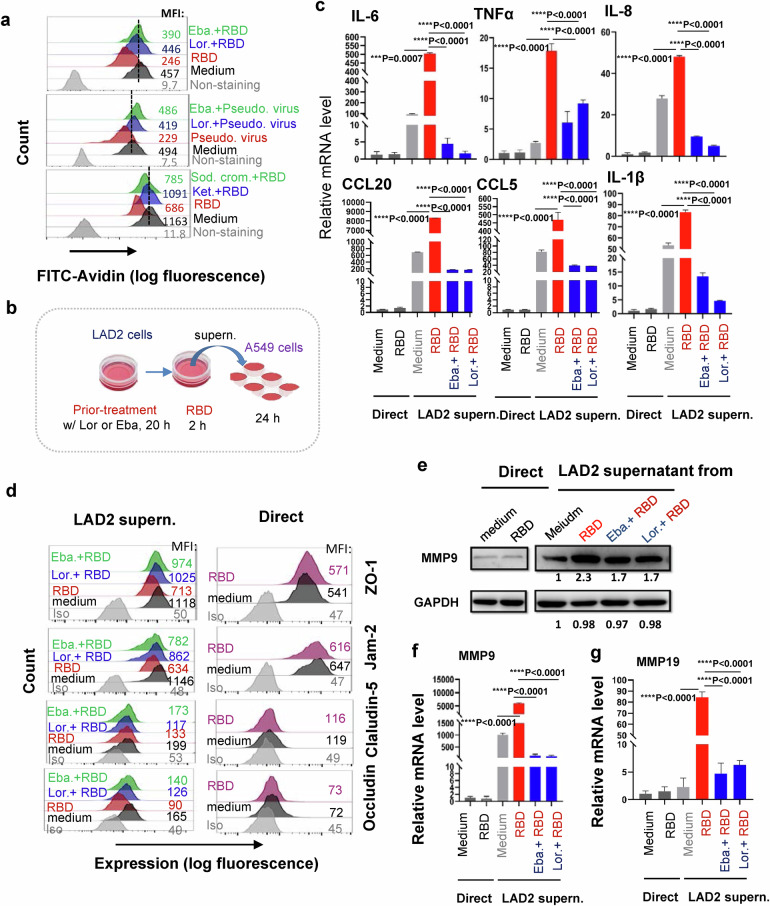
Inhibition of MC degranulation abolishes alveolar epithelial inflammation. **a** Lor., Eba., Ket., or Sod. Crom. inhibits Spike-RBD or pseudotyped lentivirus-induced LAD2 cell degranulation. Cells were prior-treated with Lor. (5 μg/mL), Eba. (3 μg/mL), Ket. (40 μg/mL), or Sod. Crom. (10 μg/mL) for 20 h, then were incubated with Spike-RBD (5 μg/ml) or Spike-pseudotyped lentivirus (5 ng p24^Gag^) at 37 °C for the 2 h, and cell degranulation was detected with Flow cytometry. **b** The illustration for treatments. LAD2 cells were prior-treated with or without Lor. (5 μg/mL) or Eba. (3 μg/mL) for 20 h, then cells were treated with Spike-RBD (5 μg/ml) for 2 h, and the culture supernatants were harvested to treat A549 cells for additional 24 h, or A549 cells were directly treated with or without Spike-RBD for 24 h, **c** the mRNA levels of IL-6, TNF-α, IL-8, CCL20, CCL5 and IL-1β were quantified with real time q(RT-) PCR, and normalized to *gapdh* mRNA, **d** the expressions of ZO-1, Jam-2, Claudin-5 and Occludin were detected by immunstaining with specific antibodies and analyzed with flow cytometry, and (**e**, **f**), the expressions of MMP9 and MMP19 in A549 cells were measured with Western blotting or real time q(RT-) PCR, and the gray intensity of gel was calculated by Image J and normalized (**e**). One representative data from 3 (**c**, **d**, **e**, **g**) or 5 (**a**, **f**) independent repeats are shown. Data are presented as mean ± SD. ***p < 0.0001 and ****p < 0.0001 are considered significant difference (**c**, **f**). MFI mean fluorescence intensity (**a**, **d**)

Our results showed that the prior-treatments of LAD2 cells with Eba., Lor., Sod. crom. or Ket., blocked Spike-RBD- and/or Spike-pseudotyped lentivirus- induced cell degranulation (Fig. 6a). Next, we thought to use these MC stabilizers to reduce the subsequent stimulation of inflammatory mediators from alveolar epithelial cells. The LAD2 cells were prior-treated with the representative MC stabilizers, the second generation of antihistamines Lor. and Eba., to block Spike-RBD-induced cell degranulation, then the cell culture supernatants were harvested to treat lung epithelial cells A549 (Fig. 6b). The direct stimulation of A549 cells with Spike-RBD did not induce obvious expression of pro-inflammatory cytokines, however the treatment of A549 cells with the LAD2/RBD supernatants induced production of extremely high level of IL-6, TNF-α, IL-8, IL-1β, CCL20 and CCL5; when the Spike-RBD-induced LAD2 cell degranulation was blocked by Lor. or Eba., the harvested supernatant lost its capacity to induce pro-inflammatory cytokines (Fig. 6c; Supplementary Fig. 6).

To confirm the observed LAD2/RBD supernatant-induced production of pro-inflammatory cytokines is not an artifact in a single cell line, the human non-small cell lung cancer cells H1299 were used for stimulation with LAD2/RBD supernatants. The same results have been gotten, as LAD2/RBD supernatants signficantly induced the expression of IL-6, IL-8, IL-1β, TNF-α in H1299 cells (Supplementary Fig. 7).

The disruption of integrity of alveolar epithelial barrier is associated with defects in the tight junction proteins. In keeping with the above transcriptome analysis data (Fig. 5h), we found that the stimulation of A549 cells with LAD2/RBD supernatants reduced the tight junction protein ZO-1, Jam-2, Claudin-5 and Occludin, but the direct stimulation with RBD proteins showed no effect; with prior-treatment of LAD2 with Lor. or Eba. to block degranulation, the harvested LAD2/RBD culture supernatants were unable to impair tight junction proteins (Fig. 6d). These suggest that specific factor or factors released into the LAD2/RBD supernatants acted on the tight junction proteins.

One of the possible candidates is Matrix Metallopeptidase 9 (MMP-9) which has significantly elevated expression and activity in COVID-19 patients.^63,64^ MMP-9 can impair the alveolar epithelial-endothelial capillary barrier by degrading the extracellular matrix, stimulate neutrophil and leukocyte migration, and promote inflammation.^65^ Keeping consistent with the above transcriptome analysis data (Fig. 5g), the LAD2/RBD supernatant-treated A549 cells had higher MMP9 expression levels, and after blocking LAD2 degranulation with Lor. or Eba., the LAD2/RBD supernatants lost its ability to induce MMP9 gene expression (Fig. 6e, f). MMP19 is another significantly up-regulated gene in our above RNA-Seq data (Fig. 5g). MMP19 is expressed in monocytes, macrophages, fibroblasts, and endothelial cells.^66^ We also confirmed that LAD2/RBD supernatants-treated A549 cells had higher MMP19 expression and the blocking of LAD2 degranulation with Lor. or Eba. lost the induction of MMP19 gene expression (Fig. 6g). Taken together, these data demonstrate that the Spike-RBD-triggered MC degranulation can further induce the production of pro-inflammatory cytokines and MMPs from A549 alveolar epithelial cells. In keeping with this mechanistic interpretation, drugs that preventing MC degranulation can reduce alveolar epithelial inflammation and protect the tight junction proteins integrity.

### MC stabilizers reduce SARS-CoV-2-induced lung inflammation and prevent lung injury in mice

**Fig. 7:**
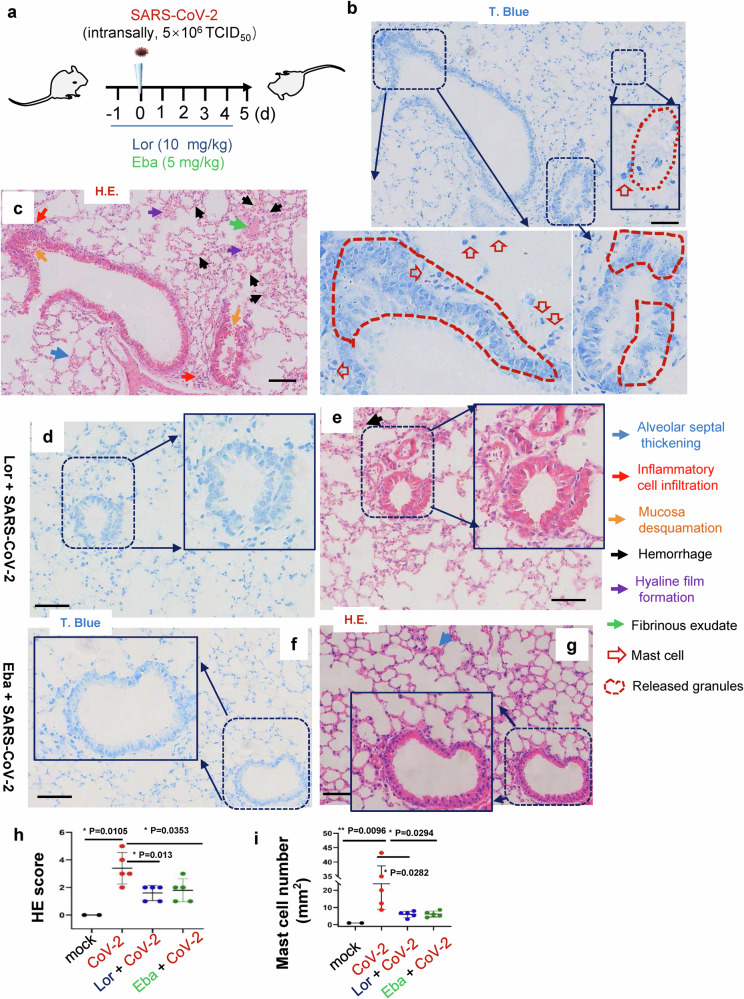
MC stabilizers prevent lung injury in mice. **a** The illustration of mice treatment. C57BL/6N-Ace2^em2(hACE2-WPRE,pgk-puro)/CCLA^ mice were prior- administered with or without Eba. (5 mg/kg) or Lor. (10 mg/kg) via i.p. 1 day before intranasal infection with SARS-CoV-2 (strain 107) at a dose of 2 × 10^6^ TCID_50_, and the Eba. and Lor. treatments were continued each day over the couse of infection. 5 mice for each treatment groups, and 2 mice without infection and drug treatment were used as the mock controls. Mice were euthanized and lung lobes were harvested for pathological analysis: Toluidine blue staining was used to observe MC degranulation, and the lung injury was observed via H.E. staining. Scale bar: 100 μm. **h** H.E. scores and (**i**) MC counts in lung section (5 dpi). *p < 0.05 and **p < 0.01 are considered significant differences

To test whether the above observation derived from in vitro study could be applied to the actual infection in vivo, we went back to the SARS-CoV-2 infection model of hACE-2 humanized mice. The C57BL/6N-Ace2^em2(hACE2-WPRE,pgk-puro)/CCLA^ were treated with Lor. (10 mg/kg) or Eba. (5 mg/kg) via i.p.1 day prior to intranasal infection with SARS-CoV-2 (strain 107) at a dose of 5 × 10^6^ TCID_50_, and then Lor. or Eba. were continued to be administered daily for 5 days until the mice were euthanized at 5 dpi (Fig. 7a).

**Fig. 8:**
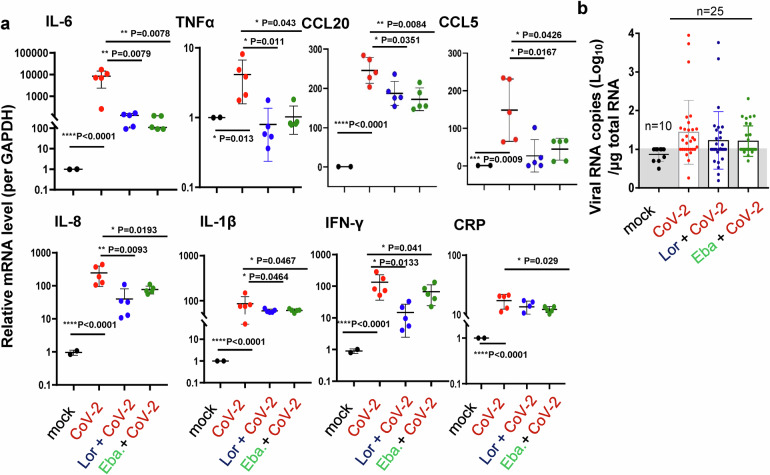
MC stabilizers reduce SARS-CoV-2-induced inflammation. C57BL/6N-Ace2^em2(hACE2-WPRE,pgk-puro)/CCLA^ mice were prior- administered with or without Eba. (5 mg/kg) or Lor. (10 mg/kg) via i.p. 1 day before intranasal infection with SARS-CoV-2 (strain 107) at a dose of 2 × 10^6^ TCID_50_, and the Eba. and Lor. treatments were continued each day over the couse of infection. 5 mice for each treatment groups, and 2 mice without infection and drug treatment were used as the mock controls. Mice were euthanized and lung lobes were harvested for analysis. **a** The mRNA levels of IL-6, TNF-α, CCL20, CCL5, IL-8, IL-1β, IFN-γ and CRP were quantified with q(RT-) PCR, and normalized to *gapdh* mRNA. **b** Viral replication was monitored by quantifying the expression of nucleocapsid gene. Data are presented as mean ± SD. *p < 0.05, **p < 0.01, ***p < 0.001 and ****p < 0.0001 are considered significant differences

In untreated controls, SARS-CoV-2 challenge induced MC accumulation in the peri-bronchus, and the robust MC degranulation was evidenced by abundant released granules in peri-bronchus and scatted in alveolar spaces (Fig. 7b). The H.E. staining of the adjacent lung sections showed lung lesions including the alveolar septal thickening, inflammatory cell infiltration, fibrinous exudate, hyaline film formation, mucosa desquamation, and hemorrhage (Fig. 7c). The administration of Lor. and Eba. blocked MC degranulation (Fig. 7d, f), and greatly reduced lung lesions (Figs. 7e, g, h, i). Additionally, the administration of these two MC stabilizers significantly dampened SARS-CoV-2-induced inflammation, as evidenced by the greatly reduced productions of IL-6, TNF-α, CCL20, CCL5, IL-8, IL-1β, IFN-γ and CRP (Fig. 8a). Viral replication was also monitored. Total RNAs were prepared from the left lobe, the right lobus superior, the right lobus medius, the right lobus inferior and the pulmonary azygos lobe of each mouse, and viral replication was measured by quantifying the expression of nucleocapsid gene. The mono-treatment with either Lor. or Eba. had not significantly reduced viral replication (Fig. 8b). Consistent with the idea that these drugs reduce SARS-CoV-2-induced lung inflammation and injury directly through preventing MC degranulation and subsequent inflammation, rather than indirectly by reducing virus load. Taken together, these data demonstrate that the administration of MC stabilizers reduces SARS-CoV-2-induced lung inflammation and prevents lung injury in mice.

## Discussion

In this study, we demonstrate a pivotal role of MCs in SARS-CoV-2-induced epithelial inflammation and lung injury in vivo and elucidate its possible mechanisms in vitro. Besides of SARS-CoV-2, the other type of Coronavirus such as HCoV-NL63 and HCoV-229E could also induce MC degranlulation. It is possible that Coronavirus-induced MC degraulation to initiate epithelial hyper-inflammation represents a common mechanism for leading to organ/tissue damage. This point is worth further investigation.

The validation of MC stabilizers Eba. and Lor. can inhibit SARS-CoV-2-induced hyper-inflammation and lung injury is highly significant, because it suggests an off-label use of MC stabilizers as immunomodulators to treat the severe cases of COVID-19. Our data in cell lines also demonstrate the blockage role of other MC stabilizers (e.g., Sodium cromoglicate and Ketotifen Fumarate) on Spike-RBD-induced MC degranulation. The further clinical testing of their beneficial role of in the setting of SARS-CoV-2 infection would be much helpful in the treatment of lung injury in COVID-19 patients.

The strategical location at mucosa makes MC being the sentinel to the exposed pathogens includuing SARS-CoV-2. By releasing the soluble factors, MCs can recruit multiple types of immune cells to beneficially or detrimentally regulate immune inflammations.^40,41^ Given our findings of SARS-CoV-2 Spike-RBD induced rapid MC degranulation, one may speculate that the MC activation and consequential inflammatory mediators release is one of the root-causes of lung inflammation and injury in COVID-19.

The phenotypes of MC degranulation-induced epithelial inflammation and lung injury in our study is concordant with the immunological and histopathological findings of COVID-19 patients and infected animals,^43,67,68^ indicating that SARS-CoV-2-induced MC degranulation is a major cause that triggers hyper-inflammation and lung injury. We find that MC degranulation induces extremely high level of productions of pro-inflammatory cytokines and chemokines including IL-6, which has been shown to be an independent risk factor for disease severity and death of COVID-19.^12,16,19–21^

Spike-RBD-triggered MC degranulation provides an important route to disrupt alveolar barrier by inducing various types of metallopeptidases. LAD2/RBD supernatant promotes the expression of MMP9 in alveolar epithelial cells, which resembles the observation of significantly increased circulating MMP9 level in COVID-19 patients.^63,64^ In acute lung injury, the released MMP9 are able to promote degradation of the alveolar-capillary barrier, through probably action on tight junction proteins.^65^

The released components of SARS-CoV-2-induced MC degranulation contain Chymase and Typtase, which can also be used to explain virus-induced permanent damage to the alveoli epithelial cells and capillary endothelial cells. In inflammation or pathological conditions, the released Chymases from activated MCs are able to amplify local angiotensin-2 concentration to induce inflammatory leucocyte recruitment and endothelial dysfunction, which would further increase pulmonary vascular permeability and cause lung injury.^69,70^ Other types of viruses such as Dengue virus has been reported to trigger the release of Chymase and Typtase to breakdown the endothelial cell tight junctions to promote vascular permeability.^71^

SARS-CoV-2 infection can shutdown the expressions of cell cycle kinases (e.g., CDK1/2/5 and AURKA, etc) and result in cell cycle arrest between S and G2 phases of the cell cycle.^67,72^ In our study, we find that Spike-RBD-triggered MC degranulation shutdown the expressions of various genes linking to the regulation of cell cycles/division. Besides, LAD2/RBD supernatants can directly suppress the expression of genes encoding the cytoskeleton/microtubule-associated proteins and myosins, suggesting MC degranulation induces the change of cytoskeleton organization. Our experimental data, again, consistent with the clinical observation that SARS-CoV-2 infection inducing changes of cytoskeletal-microtubule organization.^67,72^

It seems that the infection and replication of SARS-CoV-2 in MCs is not necessarily required for triggering degranulation, as the stimulation of Spike-RBD can trigger MC degranulation. MC cells express ACE2 receptor and are permissive for SARS-CoV-2 replication. Whether SARS-CoV-2 replication in MCs leads to the de novo synthesis of inflammatory mediators for the secondary release is worthy of further investigation. Additionally, SARS-CoV-2-induced MC degranulation may also be used to explain the disease severity in aged individuals. We find that SARS-CoV-2 induces a more robust MC degranulation in aged chRMs, which could link the severer cytokine storm and higher immune cell infiltration in aged adults and RMs.^51,68,73,74^

The limitation of our study is that the mono-administration of Eba. or Lor. shows less effect in directly suppressing viral replication in lungs of ACE2-humanized mice. A combination of MC stabilizers with antiviral drugs such as the RNA polymerase inhibitors Remdesivir and Favipiravir,^75–78^ may provide a more optimal treatment strategy for both dampening inflammation and clearing viruses at the same time.

In summary, we demonstrate that SARS-CoV-2 triggers lung MC degranulation, which induces the remodeling of various cellular signalings in human alveolar epithelial cells, particularly, MC degranulation induces the alveolar epithelial inflammation and leads to the consequent disruption of tight junction proteins; importantly, we find that the clinically used MC degranulation stabilizers Eba. and Lor. are potent agents at reducing virus-induced production of pro-inflammatory factors and preventing lung injury. Our finding uncovers a potentially novel mechanism of SARS-CoV-2 infection initiates alveolar epithelial inflammation and induces lung Injury. Significantly, our results suggest a potential off-label use of MC stabilizers as immunomodulators to treat the severe cases of COVID-19.

## Materials and methods

### Ethics statement

All animal experiments were approved by the Institutional Animal Care and Use Committee of Guangzhou Institutes of Biomedicine and Health and Kunming Institutes of Zoology, Chinese Academy of Sciences, and the first Affiliated Hospital of Guangzhou Medical University. The SARS-CoV-2 animal model experiments and protocols were also discussed explicitly and extensively with biosafety officers and facility managers. All animal experiments and wild type virus were conducted within the animal biosafety level 3 (ABSL-3) facility.

### Cells and viruses

Human mast cell LAD2 and Human non-small cell lung cancer cells H1299 were cultured in RPMI 1640 medium (Gibco) containing 10% fatal bovine serum (FBS) (Gibco) with 100 U/mL penicillin and 100 μg/mL streptomycin. Human hepatoma cell line Huh-7 was cultured in DMEM medium (Gibco) containing 10% FBS (Gibco) with 100 U/mL penicillin and 100 μg/mL streptomycin. For degranulation, LAD2 cells were grown in StemPro-34 medium (Gibco) supplemented with 100 μg/ml stem cell factor (Novoprotein), 100 μg/ml IL-6 (Novoprotein), nutrient supplement (NS) (Gibco), 100 U/ml penicillin (Invitrogen), 100 µg/ml of streptomycin (Invitrogen) and 2 mM L-Glutamine (Gibco). Adenocarcinomic human alveolar basal epithelial cells (A549) was cultured in DMEM/F12 medium (Gibco) containing 10% FBS (Gibco) with 100 U/mL penicillin and 100 μg/mL streptomycin.

The 107 strain of SARS-CoV-2 was provided by Guangdong Provincial Center for Disease Control and Prevention, Guangdong Province of China. The Coronavirus HCoV-NL63 (NR-470) and HCoV-229E (VR-740) were purchased from the American Type Culture Collection (ATCC). Pseudotyped virus was generated by EZ Trans cell transfection reagent (Life iLab, AC04L082) -mediated co-transfection of HEK293T cells with the Spike-expressing plasmid pcDNA3.1-2019-nCoV-S-IRES (strain 2019-nCoV WIV04) and pNL4-3. Luc. ΔR ΔE.^79^ These two plasmids are provided by Dr. Lu Lu (Fudan University, Shanghai, China). Harvested supernatants of transfected cells that contained viral particles were aliquoted and stored at −80 °C.

### ACE2-humanzied mice and rhesus macaques experiments

3–4 months old C57BL/6N-Ace2^em2(hACE2-WPRE,pgk-puro)/CCLA^ mice were provided by Guangzhou Institutes of Biomedicine and Health, Chinese Academy of Science.^46^ The mice were randomly assigned to each group and the ratio of females to males was 1:1. Mice (5 for each groups) were infected nasal inhalation with SARS-CoV-2 (strain 107) (5 × 10^6^ TCID_50_) for indicated times. The same amount of PBS was inoculated as the mock infection. In some mice, ebastine (5 mg/kg) or loratadine (10 mg/kg) (both from Sigma–Aldrich) was administered 1 day before infection and the treatments were continued each day over the course of infection. The lungs were collected on the day of euthanization for pathological, virological and immunological analysis.

For Ad5-hACE2-transduced BALB/c mice,^50^ specific pathogen-free 6–10 weeks old male and female (ratio = 1:1) BALB/c mice were lightly anaesthetized with isoflurane and transduced intranasally with 2.5 × 10^8^ fluorescence focus units (FFU) of Ad5-ACE2 in 75 μL DMEM. Five days post-transduction, mice were infected intranasally with 7 × 10^4^ TCID_50_ SARS-CoV-2 in a total volume of 50 μL DMEM. The same amount of DMEM was inoculated as the mock infection. The SARS-CoV-2 strains used in this experiment were isolated from COVID-19 patients in Guangzhou (Accession numbers: MT123290). At the indicated time, the lungs of mice were collected on the day of euthanization for histology analysis.

For monkey study, eight Chinese rhesus macaques (chRMs) (*Macaca mulatta*) (all male), including young group (3- to 6-year old) and aged group (17- to 19-year old), were anaesthetized by Zoletil 50 (Viabac, France) and intratracheally inoculated with SARS-CoV-2 (virus stain107) (1 × 10^7^ TCID_50_) in a 2 mL volume by bronchoscope. The animals were euthanized at 7 or 15 dpi and the lung lobes were collected for histology analysis.^51^

### Histology

Animal’s lungs were fixed in zinc formalin. For routine histology, tissue sections (~4 μm each) were stained with Hematoxylin and Eosin or Toluidine blue. The sections were analyzed with the Motic Digital Scanning apparatus (BA600Mot-4C-VM). The pathological score was assessed according to the degree of lung tissue lesions including alveolar septal thickening, hemorrhage, inflammatory cells infiltration, and consolidation. The semiquantitative assessment were performed as follows,^46^ 0: no alveolar septal thickening; 1: alveolar septal thickening was very mild, the area of alveolar septal thickening, hemorrhage and inflammatory cells infiltration was less than 10%; 2: when alveolar septal thickening was mild, the area of alveolar septal thickening, hemorrhage and inflammatory cells infiltration was 10–25%; 3: when alveolar septal thickening was moderate, the area of alveolar septal thickening, hemorrhage, inflammatory cells infiltration, hyaline film formation, mucosa desquamation and fibrinous exudate was 25–50%; 4: when alveolar septal thickening was marked, the area of alveolar septal thickening, hemorrhage, inflammatory cells infiltration, hyaline film formation, mucosa desquamation and fibrinous exudate was 50–75%; 5: when alveolar septal thickening was very marked, the area of alveolar septal thickening, hemorrhage, inflammatory cells infiltration, hyaline film formation, mucosa desquamation and fibrinous exudate was greater than 75%.

### Spike-RBD protein binds to LAD2 cells

LAD2 cells (3 × 10^5^) were incubated with Spike-RBD protein (5 μg/mL, Genscript, Z03483) in adherent buffer (1 mM CaCl_2_, 2 mM MgCl_2_ and 5% BSA, pH 7.4) for 1 h at 4 °C. The cells were then fixed with 4% paraformaldehyde (Sigma–Aldrich) for 30 min at room temperature and stained with anti-His-tag antibodies (Abmart, M30111S). Subsequently, the cells were stained with goat anti-mouse Alexla Fluor 488-conjugated secondary antibodies (Invitrogen, A11001), and were detected with flow cytometry (BD Accuri C6) and analyzed with the FlowJo 7.6.1 software. In some experiments, LAD2 cells were prior-blocked with anti-ACE2 antibody (5 μg/mL, R&D Systems, AF933) for 1 h at 37 °C before the incubation with Spike-RBD protein.

### LAD2 cell degranulation

LAD2 cells (3 × 10^5^) were exposed to Spike-RBD protein (Genscript) (5 μg/mL), nucleocapsid protein (5 μg/mL), SARS-CoV-2 (M.O.I. = 1) (strain 2019-nCoV WIV04), or HCoV-NL63 (ATCC, NR-470) and HCoV-229E (ATCC, VR-740) (M.O.I. = 1) for the indicated times. Mast cell degranulation activator compound 48/80 (C48/80) (4 μg/ml) (Sigma, C2313) was used as the control. Cells were fixed with 4% paraformaldehyde (Sigma–Aldrich) at room temperature for 30 min, and washed 3 times with PBS, then cells were incubated with anti-avidin-FITC (500 ng/mL, Invitrogen, A821) which was diluted in permeabilized buffer (1% FBS and 0.2% Triton X-100 in PBS) at 4 °C for 1 h. After washing, cells were detected with BD Accuri C6 and analyzed with FlowJo. In some experiments, Loratadine (5 μg/mL, Selleck), Ebastine (3 μg/mL, Selleck), Ketotifen Fumarate (40 μg/mL, Yuanye Biology, China, S46226), or Sodium cromoglycate (10 μg/mL, Sigma, 15826-37-6) was used to prior-treat cells for 20 h before stimulation with Spike-RBD protein. The LAD2 cell culture supernatants were harvested for quantifying the released components of Chymase and Tryptase with ELISA kits according the manufacturer’s instructions instruments (Lunchangshuo Biotech, Tryptase: SU-B10563; Chymase: SU-B16617).

### Inflammatory cytokine assay

A549 cells (3 × 10^5^) were treated with LAD2 culture supernatant (250 μL) for 24 h, then cells were harvested. The cytokines were determined either by quantifying the production of mRNAs or intracellular immuno-staining with specific antibodies. For immuno-staining assay, A549 cells were added leukocyte activation cocktail containing BD GolgiPlug (BD, 550583) and cultured for 6 h. Following activation, cells were washed with FACS buffer. BD Cytofix/Cytoperm solution (BD, 554722) was used for the simultaneous fixation and permeabilization of cells for 20 min at 4 °C before intracellular cytokine staining. Antibodies diluted in Perm/Wash buffer was added and cells were further incubated at 4 °C for overnight. After washing, cells were resuspended in FACS buffer to flow cytometric analysis (BD LSRFortessa). Cytokine antibodies against the flowing markers were used: Alexa Fluor 647-IL-1β (Biolegend, JK1B-1), PE-IL-6 (BD, MQ2-6A3), BV421-IL-8 (BD, G265-8).

### Real time (RT-) PCR

Total RNAs from cells were extracted by using TRIzol Reagent (Invitrogen) and then reverse transcribed into cDNA with synthesis Kit (TOYOBO, FSQ-301), according to the manufacturer’s instructions. Real-time PCR was carried out by using the SYBR qPCR Mix (Genestar, A33–101) with the following thermal cycling conditions: initial denaturation at 95 °C for 2 min, amplification with 40 cycles of denaturation at 95 °C for 15 s, primer annealing at 60 °C for 15 s, and extension at 72 °C for 30 s. The data were analyzed by SYBR green-based semi-quantification and normalized with GAPDH. Real-time PCR was performed on the Bio-Rad CFX96 Real-Time PCR system. The mice extracted RNAs were used to measure the copies of nucleocapsid gene SARS-CoV-2 using THUNDERBIRD Probe One-step qRT-PCR Kit (Toyobo). The standard samples were purchased from the National Institute of Metrology of China. The primers and probes for (RT-) PCR were listed in Supplementary Table 1.

### Western blotting

LAD2 or A549 cells were lysed for 1 h at 4 °C in lysis buffer (Beyotime). After centrifugation for 10 min at 12,000 g, the supernatant was boiled in reducing SDS sample loading buffer and analyzed by SDS-PAGE. The anti-ACE2 antibody (Abcam, EPR4435), anti-MMP9 antibody (signal antibody, JA80–73), anti-GAPDH antibody (Abcam, ab82633), and the horseradish peroxidase-conjugated secondary antibody were used in Western blotting.

### Flow cytometry

The expression of ACE2 in LAD2 was determined by immunostaining with PE-labeled rabbit anti-ACE2 (Bioss, bs-1004R) and detecting with flow cytometry (BD Accuri C6). For detecting tight junction proteins ZO-1, Occludin, Claudin-5 and JAM2 in A549 cells, cells were blocked with 5% BSA in PBS for 1 h at room temperature then incubated with primary antibodies for 2 h at 4 °C. Primary antibodies against ZO-1 (Invitrogen, 402200), Occludin (Invitrogen, OC-3F10), Claudin-5 (Invitrogen, 4C3C2) and JAM-2 (Abcam, EPR2489), were used. A permeabilizing agent (1% FBS and 0.2% Triton X-100 in PBS) was used for ZO-1 intracellular staining. Cells were washed with FACS buffer and then incubated with Alexa Flour 488-labeled goat anti-rabbit or goat anti-mouse IgG (Invitrogen, A11034; Invitrogen, A11001) for 1 h at 4 °C, then cells were analyzed with flow cytometry (BD Accuri C6).

### RNA sequencing and data analysis

A549 cells were treated with LAD2 cell culture supernatants for 24 h. Total RNAs were extracted using Trizol (Invitrogen) according to the manufacturer’s protocol, and ribosomal RNA removed using QIAseq FastSelect-rRNA HMR Kits (QIAGEN, Germany). Fragmented RNAs (average length ~200 bp) were subjected to first strand and second strand cDNA synthesis, followed by adaptor ligation and enrichment with a low-cycle according to the instructions of NEBNext UltraTM RNA Library Prep Kit for Illumina (NEB, USA). The purified library products were evaluated using the Agilent 2200 TapeStation and Qubit2.0 (Life Technologies, USA). The libraries were paired-end sequenced (PE150, Sequencing reads were 150 bp) at Guangzhou RiboBio Co., Ltd. (Guangzhou, China) using Illumina HiSeq 3000 platform.

Raw RNA sequencing (RNA-seq) reads were filtered using Trimmomatic v0.36. The filtered reads were mapped to the human (hg38) reference genomes using HISAT v2.1 with corresponding gene annotations (GRCh38.p13) with default settings. Total counts per mapped gene were determined using featureCounts function in SubReads package v1.5.3 with default parameter. Next, counts matrix obtained from featureCounts was used as input for differential expression gene analysis with the bioconductor package DESeq2 v1.26 in Rv4.0. Gene counts more than 5 reads in a single sample or more than 50 total reads across all samples were retained for further analysis. Filtered counts matrix was normalized using the DESeq2 method to remove the library-specific artifacts. Principal component analysis was based on global transcriptome data using the build-in function *prcomp* in R software. The genes with log2 fold change >1 or < **−**1 and adjusted *p* < 0.05 corrected for multiple testing using the Benjamini–Hochberg method were considered significant. Transcription-factor enrichment analysis and functional enrichment analysis was performed using Metascape server tool^80^ (https://metascape.org/gp/index.html#/main/step1). Gene set enrichment analysis (GSEA) used the R package clusterProfiler v3.18.1. Protein–protein interaction (PPI) networks of DEGs were built using STRING v11 with a confidence score threshold of 0.7 and visualized with Cytoscape v3.8.1.

### Statistical analysis

Graphpad Prism 8.0 was used for statistical analysis. For intra-group direct comparisons, Student’s unpaired two-tailed *t* test was performed to analyze significant differences. For comparisons of multiple groups, one-way ANOVAs were performed.

## Supplementary information

Supplementary Figures and Table

## Data Availability

All raw RNA-seq data used in this study have been deposited at the https://www.ncbi.nlm.nih.gov/bioproject/PRJNA741047.
