# Correction: SARS-CoV-2-triggered mast cell rapid degranulation induces alveolar epithelial inflammation and lung injury

**DOI:** 10.1038/s41392-025-02465-8

**Published:** 2025-10-31

**Authors:** Meng-Li Wu, Feng-Liang Liu, Jing Sun, Xin Li, Xiao-Yan He, Hong-Yi Zheng, Yan-Heng Zhou, Qihong Yan, Ling Chen, Guo-Ying Yu, Junbiao Chang, Xia Jin, Jincun Zhao, Xin-Wen Chen, Yong-Tang Zheng, Jian-Hua Wang

**Affiliations:** 1 College of Life Science, Henan Normal University, Xinxiang, China; 2 Key Laboratory of Animal Models and Human Disease Mechanisms of the Chinese Academy of Sciences, Kunming Institute of Zoology, Chinese Academy of Sciences, Kunming, China; 3 State Key Laboratory of Respiratory Disease, National Clinical Research Center for Respiratory Disease, Guangzhou Institute of Respiratory Health, The First Affiliated Hospital of Guangzhou Medical University, Guangzhou, Guangdong, China; 4 Guangzhou Institutes of Biomedicine and Health, Chinese Academy of Sciences, Guangzhou, China; 5 Bioland Laboratory, Guangzhou Regenerative Medicine and Health Guangdong Laboratory, Guangzhou, China; 6 Shanghai Public Health Clinical Center Affiliated to Fudan University, Shanghai, China; 7 University of Chinese Academy of Sciences, Beijing, China

Correction to: *Signal Transduction and Targeted Therapy* 10.1038/s41392-021-00849-0, published online 17 December 2021

After the publication of this review article^1^, a miscitation was identified in the Introduction part. In the sentence “Besides, US FDA has approved and authorized two mRNA vaccines (Pfizer-BioNTech, Moderna) and one recombinant adenovirus-26 vaccine (Janssen, Ad26.COV2.S) for population use,5 and globally more than 100 candidate SARS-CoV-2 vaccines are under development 6”, the reference 5 bearing no relevance to vaccine approvals was cited by an inadvertent mistake. Therefore, this reference should be removed. We apologize for any inconvenience this has caused. The key findings of the article are not affected by this correction.

The original article has been corrected.
