# Supplementary material for: SARS-CoV-2-triggered mast cell rapid degranulation induces alveolar epithelial inflammation and lung injury
Source: Signal Transduct Target Ther. 2021 Dec 17;6:428. doi: 10.1038/s41392-021-00849-0 (PMC8677926; doi:10.1038/s41392-021-00849-0)
Supplement: Supplementary file 1 — Supplementary Figures and Table [file 41392_2021_849_MOESM1_ESM.pdf]

Supplementary Materials for

SARS-CoV-2-Triggered Mast Cell Rapid Degranulation Induces Alveolar Epithelial Inflammation and Lung Injury

Meng-Li Wu[1], Feng-Liang Liu[2], Jing Sun[3], Xin Li[4], Xiao-Yan He[2,7], Hong-Yi Zheng[2], Yan-Heng Zhou[4], Qihong Yan[4], Ling Chen[4], Guo-Ying Yu[1], Junbiao Chang[1], Xia Jin[5], Jincun Zhao[3], Xin-Wen Chen [4,7 *], Yong-Tang Zheng[2,7 *], Jian-Hua Wang [4, 6, *]

[*]**Correspondence to**:

J.-H, Wang, Email: wang_jianhua@gibh.ac.cn

Y.-T, Zheng, Email: zhengyt@mail.kiz.ac.cn

X.-W, Chen, Email: Chen_xinwen@gibh.ac.cn

This PDF file includes:

Figure S1 to S7

Table S1

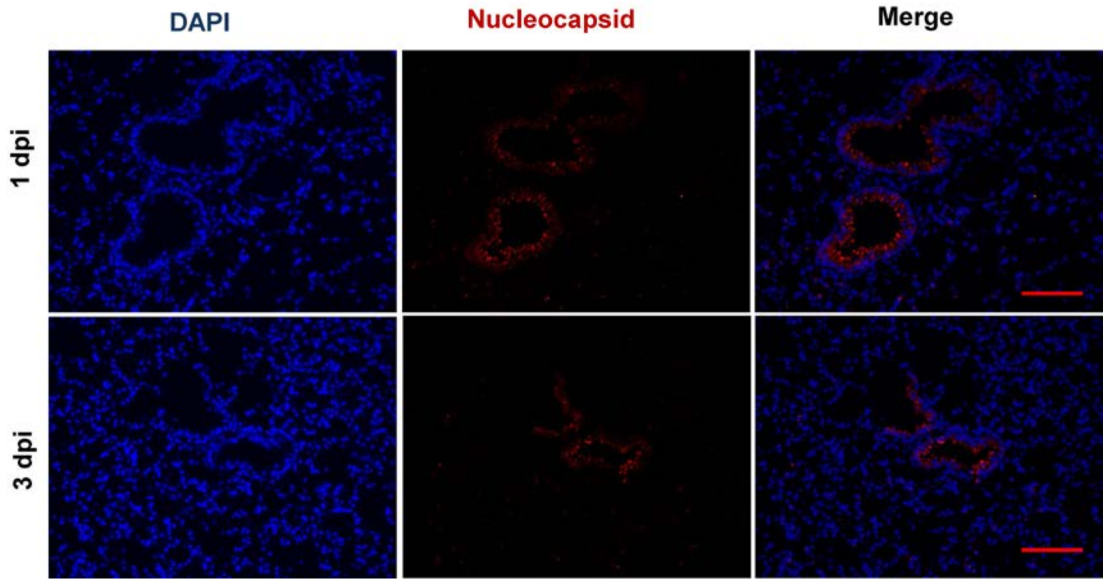

Figure S1. Virus distribution in SARS-CoV-2-infected humanized mice. C57BL/6N-Ace2$^{em2(hACE2\text{-}WPRE,pgk\text{-}puro)/CCLA}$ mice were intranasally infected with SARS-CoV-2 (strain 107) at a dose of 2 $\times 10^6$ $TCID_{50}$, and were euthanized at the 1 dpi and 3 dpi. Lung sections were stained with anti-nucleocapsid antibody (red) and DAPI (blue).

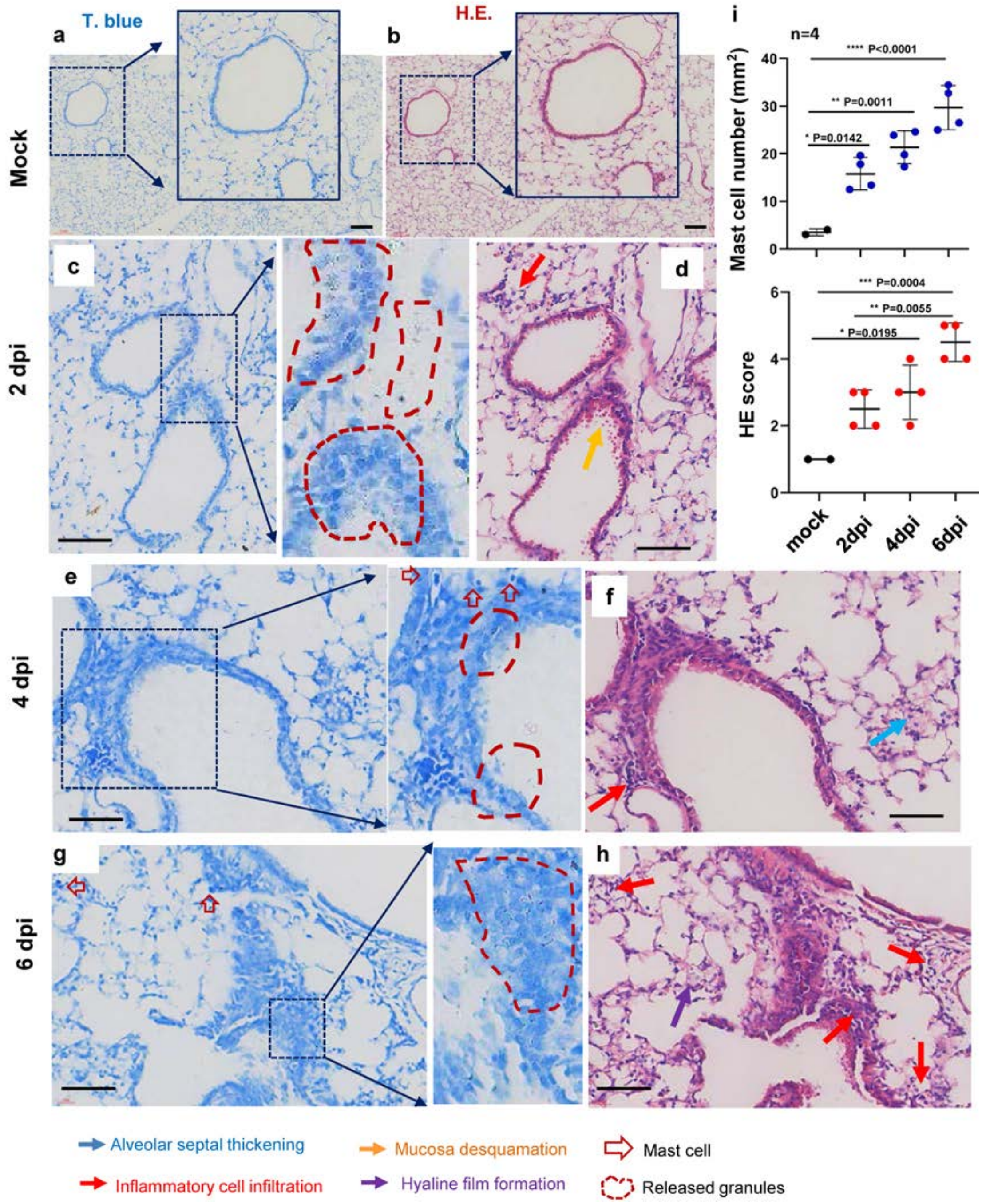

Figure S2. SARS-CoV-2 induces mast cell degranulation and lung injury in Ad5-hACE2-transduced BALB/c mice.

Five Mice were transducted with 2.5×10^{8} FFU of Ad5-hACE2 in 75 mL of DMEM intranasally, 5 days later, mice received 7×10^{4} $TCID_{50}$ of SARS-CoV-2. Two mice were used as the mock-infections. The lungs were collected at 2 dpi, 4 dpi, and 6 dpi. Toluidine blue staining was used to observe MCs and their

degranulation (a, c, e and g), and the lung injury was observed by H.E. staining (b, d, f, h). Scale bar: 100 μm. (i)The pathological score was assessed according to the degree of lung tissue lesions and MC count in lung sections was calculated. *p<0.05, **p<0.01, ***p<0.001, and **** p<0.0001 are considered significant differences.

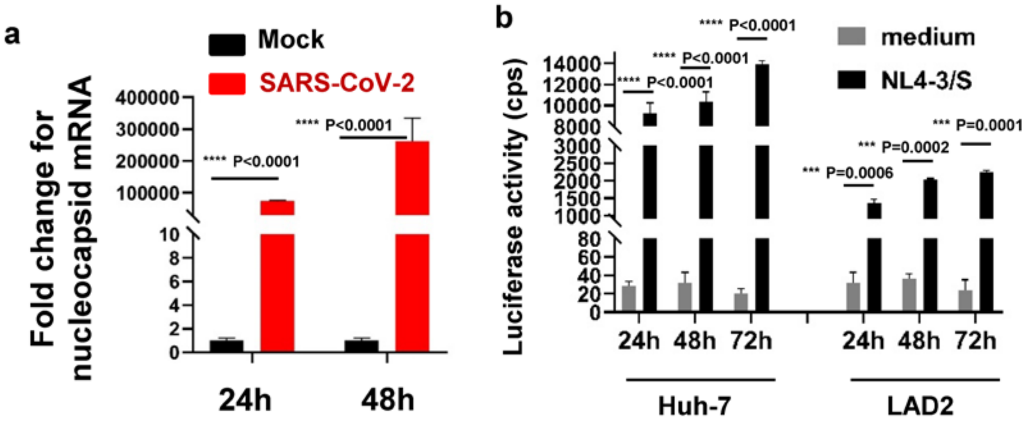

Figure S3. SARS-CoV-2 infection of LAD2 cells.

(a) LAD2 cells were infected with SARS-CoV-2 (strain 2019-nCoV WIV04) (M.O.I. =1) for the indiated time, and the cellular levels of nucleocapsid mRNA were quantified with q(RT-)PCR. Data are normalized to β-actin mRNA and expressed as –ΔCt values. (b) LAD2 and Huh-7 cells (1×10^5) were infected with SARS-CoV-2 Spike-pseudotyped lentivirus (NL4-4/S) (5 ng $p24^{gag}$) for 72 h, and viral infection was determined by measuring the luciferase activity. One representative data from 3 independent repeats are shown (a, b). Data are presented as mean ± SD. ***p<0.001 and **** p<0.0001 are considered significant differences.

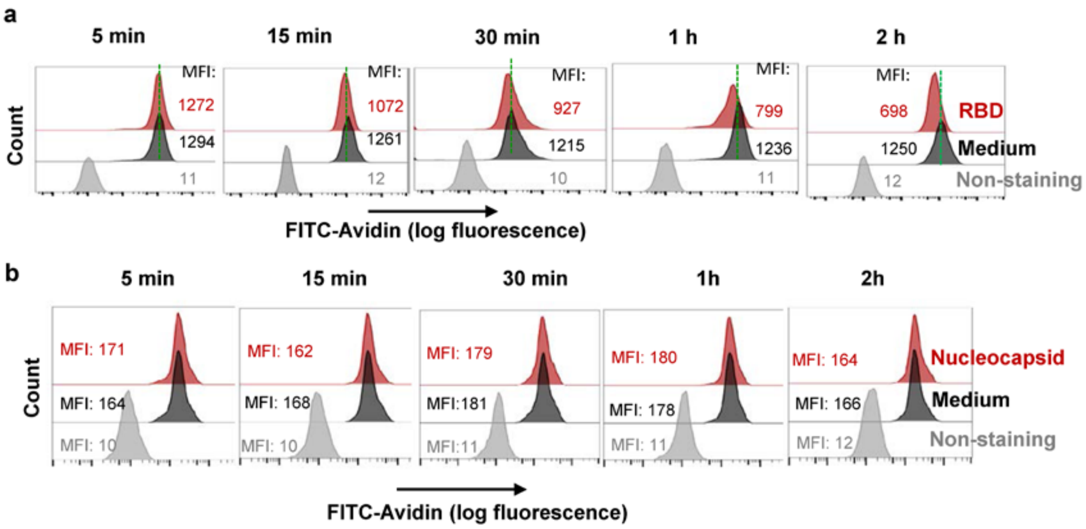

Figure S4. Assay for LAD2 cell degranulation.

LAD2 cells were treated with or without Spike-RBD (a) or nucleocapsid protein (b) (5 μg/ml each) of SARS-CoV-2 at 37°C for the indicated time, then cells were permeabilized and immunostained with anti-avidin-FITC at 4°C for 1 h, and analyzed with flow cytometry. One representative result from 7 repeats is shown. MFI: mean fluorescence intensity (a, b).

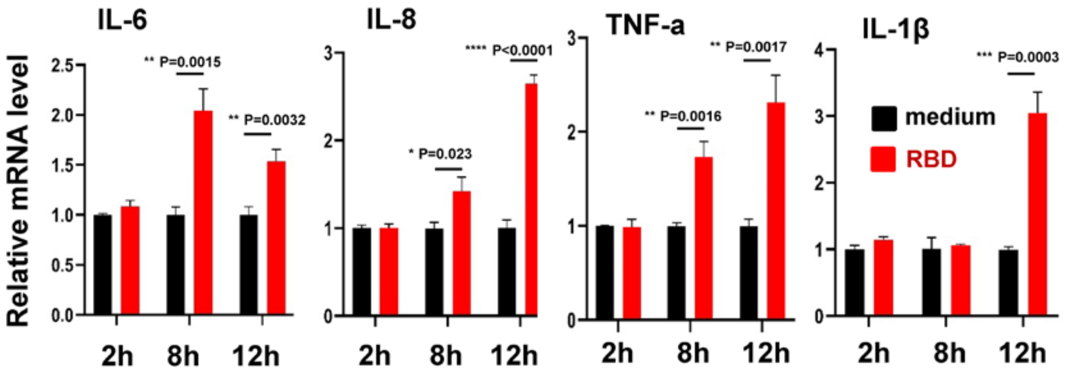

Figure S5. Cytokine profile in LAD2 cells.

LAD2 cells were treated with or without Spike-RBD (5 μg/ml) at 37°C for the indicated time, and the mRNA levels of IL-6, IL-8, IL-1β and TNF-α were quantified with q(RT-) PCR, and normalized to *gapdh* mRNA. One representative result from 3 repeats is shown. Data are presented as mean ± SD. * $p<0.05$, ** $p<0.01$, *** $p<0.001$ and ****$p<0.0001$ are considered significant differences.

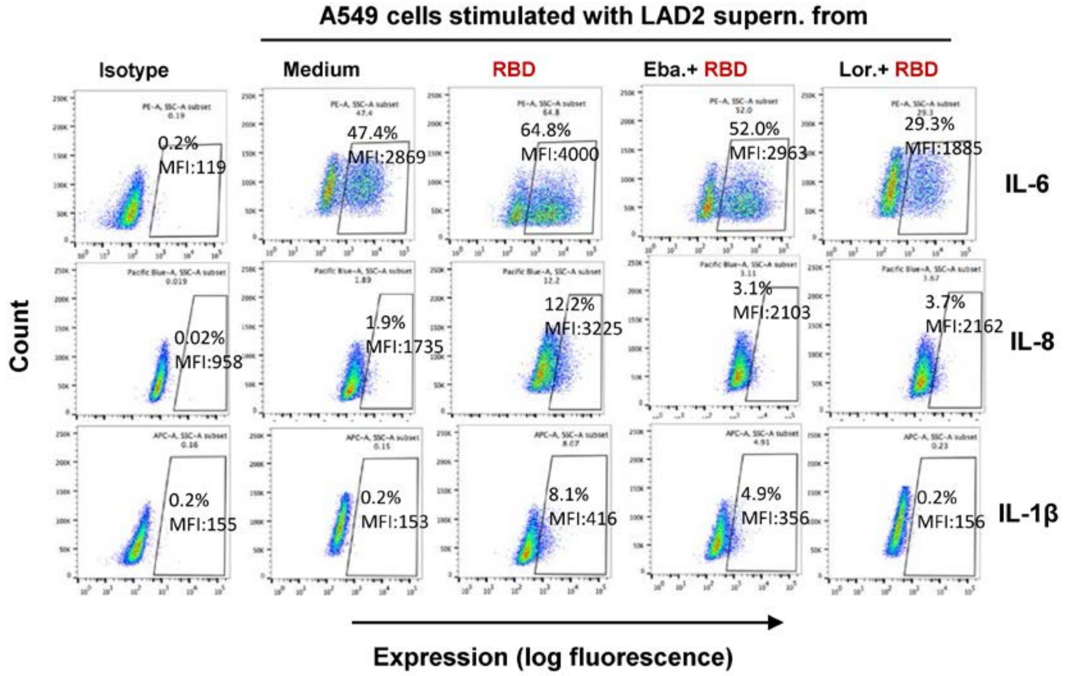

Figure S6. Cytokine productions in A549 cells.

LAD2 cells were prior-treated with or without Lor. (5 μg/mL) or Eba. (3 μg/mL) for 20 h, then cells were treated with Spike-RBD (5 μg/ml) for 2 h, and the culture supernatants were harvested to treat A549 cells for additional 24 h, or A549 cells were directly treated with or without Spike-RBD for 24 h. The productions of IL-6, IL-8, and IL-1β were measured with intracellular immunostaining with specific antibodies. One representative result from 3 repeats is shown. MFI: mean fluorescence intensity.

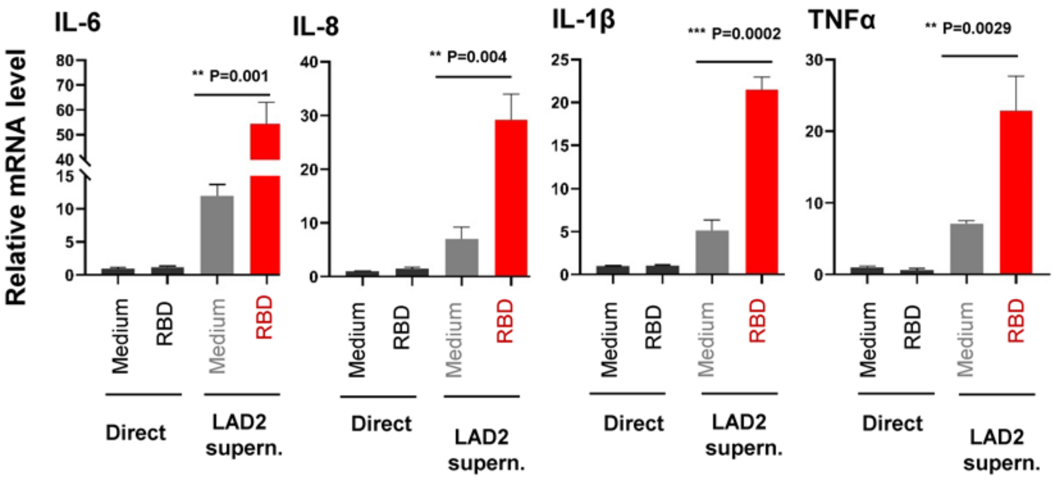

Figure S7. Cytokine expression in H1299 cells.

LAD2 cells were treated with Spike-RBD (5 μg/ml) for 2 h, and the culture supernatants were harvested to treat H1299 cells for additional 24 h, and the mRNA levels of IL-6, TNF-α, IL-8 and IL-1β were quantified with real time q(RT-)PCR, and normalized to *gapdh* mRNA. One representative result from 3 repeats is shown. Data are presented as mean ± SD. ** $p<0.01$ and *** $p<0.001$ are considered significant differences.

**Human IL-6**	Forward:	5′-CAG ACA GCC ACT CAC CTC TTC AG-3′
	Reverse	5′-CAG CCA TCT TTG GAA GGT TCA G-3′
**Human TNF-α**	Forward:	5′-CCC AGG CAG TCA GAT CAT CTT C-3′
	Reverse	5′-GTG AGG AGC ACA TGG GTG GAG-3′
**Human IL-1β**	Forward:	5′-CGT CAG TTG TTG TGG CCA TGG A-3′
	Reverse	5′- GAG CGT GCA GTT CAG TGA TCG TA-3′
**Human IL-8**	Forward:	5′-CTG ATT TCT GCA GCT CTG TGT GA-3′
	Reverse	5′-GGT CCA GAC AGA GCT CTC TTC CA-3
**Human CCL20**	Forward:	5′-GCT GCT TTG ATG TCA GTG CT-3′
	Reverse	5′-TGT CAC AGC CTT CAT TGG C-3′
**Human CCL5**	Forward:	5′-ACC ACA CCC TGC TGC TTT G-3′
	Reverse	5′-GCG GTT CTT TCG GGT GAC A-3′
**Human MMP9**	Forward:	5′-CCT TCT ACG GCC ACT ACT GTG C-3′
	Reverse	5′-GCC AGT ACT TCC CAT CCT TGA AC-3′
**Human MMP19**	Forward:	5′ -GCA ATG TGGC TCC CTT GAC-3′
	Reverse	5′ -TCA GTC CAG AAC TCG TCT TCG-3′
**Human GAPDH**	Forward:	5′-ATC CCA TCA CCA TCT TCC AGG-3′
	Reverse	5′-CCT TCT CCA TGG TGG TGA AGA C-3′
**Mouse IL-6**	Forward:	5′-CTT CCA TCC AGT TGC CTT CTT G-3′
	Reverse	5′-AAT TAA GCC TCC GAC TTG TGA AG-3′
**Mouse TNF-α**	Forward:	5′-CAG ACC CTC ACA CTC AGA TCA TCT-3′
	Reverse	5′- CCT CCA CTT GGT GGT TTG CTA-3′
**Mouse IL-1β**	Forward:	5′- CTT TCA GAG GCC AGA GAG TCC-3′
	Reverse	5′- TCC CTG TAG TGA CAG CAC CT3′
**Mouse IL-8**	Forward:	5′-CGG CAA TGA AGC TTC TGT AT-3′
	Reverse	5′-CCT TGA AAC TCT TTG CCT CA-3′
**Mouse INFγ**	Forward:	5′- ATG AAC GCT ACA CAC TGC ATC-3′
	Reverse	5′- CCA TCC TTT TGC CAG TTC CTC-3′
**Mouse CRF**	Forward:	5′- CAG AGA TTC CTG AGG CTC CAA CA-3′
	Reverse	5′- AGT CAC CGC CAT ACG AGT CCT G-3′
**Mouse CCL20**	Forward:	5′-AAG ACA GAT GGC CGA TGA AG-3′
	Reverse	5′-AGG TTC ACA GCC CTT TTC AC-3′
**Mouse CCL5**	Forward:	5′-GTG CCC ACG TCA AGG AGT AT-3′
	Reverse	5′-GGG AAG CTA TAC AGG GTC A-3′
**Mouse GAPDH**	Forward:	5′- TGC ACC ACC AAC TGC TTA G-3′
	Reverse	5′- GAT GCA GGG ATG ATG TTC-3′
**SARS-CoV-2 nucleocapsid**	Forward:	5′-GGG GAA CTT CTC CTG CTA GAA T-3′
	Reverse	5′-CAG ACA TTT TGC TCT CAA GCT G-3′
	TaqMan probe	5′-FAM-TTG CTG CTG CTT GAC AGA TT-TAMRA-3′

Table S1. The primers and probes for (RT-) PCR
